# A Quest for a Universal Plasma-Derived Antivenom Against All Elapid Neurotoxic Snake Venoms

**DOI:** 10.3389/fimmu.2021.668328

**Published:** 2021-04-23

**Authors:** Kavi Ratanabanangkoon

**Affiliations:** Department of Microbiology, Faculty of Science, Mahidol University, Bangkok, Thailand

**Keywords:** universal antivenom, pan-specific antivenoms, elapid snakes, neurotoxic venoms, plasma-derived antivenoms, diverse toxin repertoire, immunization strategy, low dose low volume multi-site immunization

## Abstract

This review describes the research aimed at the development of universal antivenom against elapid neurotoxic snake venoms. The antivenoms produced in Thailand in the 1980s were of low potency, especially against the elapid venoms. This was thought to be due to the low immunogenicity of the α-neurotoxins, which are the most lethal toxins in these venoms. Comparisons of various α-neurotoxin conjugates and polymers, and also different immunological adjuvants, showed that the adjuvant used is the major determinant in the antibody response in horses. The potent Freund’s adjuvant was not used due to its severe local side-effect in horses. Therefore, a novel immunization protocol termed ‘low dose, low volume multi-site’ was developed for use in horses. This immunization protocol has led to the production of highly potent monospecific antivenoms against several elapid and viperid venoms, and two potent polyspecific antivenoms, one against 4 neurotoxic and another against 3 hematotoxic venoms. The immunization protocol has also led to other improvements in antivenom production including: several fold increases in antiserum potency, a reduction in the time required to reach therapeutically useful antibody titers, a 90% reduction in the amount of venom used, and 100% of the horses responding to the immunization program. This development is partly responsible for significant decrease in the Thailand’s annual snakebite death toll from a few dozens to mostly nil in recent years. Finally, a simple and novel immunization strategy, using a ‘diverse toxin repertoire’ composed of numerous elapid toxin fractions as immunogen, was proposed and tested. This immunization procedure has resulted in the successful production of a widely paraspecific antiserum against at least 36 neurotoxic venoms of 28 species encompassing 10 genera and from 20 countries on four continents, and possibly against all elapid venoms with α-neurotoxins as the lethal toxins. These results indicate that, with optimizations of the composition of the ‘diverse toxin repertoire’, the immunization scheme and antibody fractionation to increase the antivenom neutralizing potency, an effective universal antivenom against the neurotoxic elapid snakes of the world can be produced.

## Introduction

Snakebite envenomation is an important medical problem in many tropical countries (1). It has been estimated that snake bites are responsible for about 400 000 disabilities with 138,000 deaths annually (2).WHO has designated this problem as a Category A most neglected tropical disease and it has spearheaded efforts to reduce the deaths and disabilities inflicted by snakebites by half in 2030 (3, 4).

The most effective treatment for snakebite envenomation is the timely administration of safe and effective antivenom (AV). Currently available AVs are plasma-derived preparations (PDAVs) produced in large animals e.g. horses, sheep etc. Despite their demonstrated efficacy, current antivenoms have a number of drawbacks, including their low titer against relevant toxins of low immunogenicity. Thus they must be administered in large doses, which contribute to the high cost of treatment and the risk of adverse reactions. Furthermore, AVs are specific in that they are mostly effective against the venom(s) used in the immunization. Thus, despite the existence of cross-reactivity of antivenoms against some heterologous venoms, there are many instances where such cross-neutralization does not occur (5–7).

The immunological specificity makes it often necessary to identify the culprit snake before specific AV treatment. Also, with geographical variation within a given snake species, AV may be effective only against the venoms of certain snakes in specific countries or regions. Consequently, most AVs are produced in small volumes for use in a limited geographical area and thus the cost is high and often unaffordable to the snake bite victims which mostly reside in low-income countries (8). Another often cited drawback of PDAV is the heterologous source of plasma which could contribute to adverse reactions, such as immediate type hypersensitivity and serum sickness, in patients. However, when antivenoms are manufactured following good manufacturing practices (GMPs) and are composed by highly purified immunoglobulins or immunoglobulin fragments, their safety profile is adequate (1). Thus, there are several issues regarding the use of PDAV in the treatment of snakebite victims and attempts are being made to improve effectiveness, reduce the number of adverse reactions and develop cheaper alternatives (9).

Because of the shortcomings of the PDAV mentioned above, there is a growing interest in the development of ‘new generation antivenoms’ using new state-of-the-arts approaches (10, 11). Examples of these novel alternatives are: human monoclonal antibody with different types of antibody formats i.e. whole IgG, single-chain variable fragments (scFvs), antigen binding fragments (Fabs and F(ab’)_2_) (12), oligonucleotide aptamers (13), inhibitors of enzymatic toxins (14), inhibitors of phospholipases A_2_ (15), inhibitors of snake venom proteases (16, 17), inhibitors of hyaluronidase (18), metal chelators (19–21) and neuronal acetylcholine receptor (nAChR) mimetics (22). All of these represent promising and interesting alternative therapeutic modalities to improve or replace PDAV, increase effectiveness, cause less adverse effects, and be cheaper to produce.

Whether the AV is plasma derived or synthetic, ideally it should be effective against the venom(s) used as immunogens and have an adequate safety profile. Moreover, the production cost, which includes the amount and cost of venom(s) and immunological adjuvant, should be low. The preparation of immunogen should be simple so that the production process can be easily carried out by manufacturers in developing countries. Importantly, the antivenom should be inexpensive and affordable to the snake bite victims. Furthermore, the AV should exhibit wide para-specific so as to be effective against other snake venoms producing a similar syndrome, and preferably against snakes in a wide geographic area or, even better, worldwide. If ‘universal’ AV can be produced and used in envenomations caused by numerous snakes, like the anti-rabies or anti-tetanus antitoxins, it can then be produced in large volumes, with the consequent reduction in production costs.

While the studies on these ‘new generation AVs’ are under active research, the production of conventional PDAV continues, and the products are currently being used to save countless lives worldwide. Moreover, some of the ‘new generation AVs’ may face hurdles due to the high cost and lack of information on venom and AV pharmacology, and the need to develop clinical trials to validate their use (23). Thus, the production of the ‘new generation AVs’ could take some time since none has progressed to clinical trials. Therefore, it is important that any feasible improvements to conventional PDAV should be explored and exploited. In the short term, it is relevant to point out that any simple improvements, quantitatively and/or qualitatively, to conventional PVAV production would be of immediate benefit to snakebite victims. Hence, the improvement of currently available antivenoms is a priority in the WHO strategy for reducing the impact of snakebite envenomation (3).

In this regard, one potentially fruitful adaptation to PDAV at present is to make possible the production of pan-specific or universal PDAV against the neurotoxic snakes. This is a line of research that we have carried out over the past several years and it constitutes the main topic of discussion of this review.

## Previous Problems Encountered in the Production of PDAV

AVs were first produced in Thailand by the Thai Red Cross Society at Queen Saovabha Memorial Institute (QSMI) in 1916, only 21 years after Albert Calmette’s groundbreaking report in 1894. The production process had changed little even until the 1980s. AVs available then were of low potency (24) and were in short supply and sufficient for probably less than half of the demand in the country. This was due to several problems. Firstly, a low percentage of horses responded to the immunization, especially so for the horses injected with neurotoxic elapid venoms (less than 20% of them responded). Secondly, a long immunization period was needed to reach acceptable antibody titers (6-8 months). Finally, the immunization program usually required a large amount of venom(s). In some cases up to 150 mg venom per horse was required. These problems were encountered not only in Thailand but likely in other antivenom producers in Asia and elsewhere.

### Immunogenicity of the Elapid Postsynaptic Neurotoxins and the Role of Immunological Adjuvant

The low potency of the anti-elapid antivenoms was thought to be due to the high toxicity and the low immunogenicity of the major lethal toxins of elapid venoms. These toxins are mostly α-neurotoxins, which are low molecular mass proteins of about 6-7 kDa (25). They bind quasi-irreversibly to the alpha subunits of the nicotinic acetylcholine receptor (nAChR) leading to blockage of neuro-muscular transmission at the muscle motor endplates (26). The high toxicity of the venoms limited the immunization doses previously believed to be required for a high antibody response (27). Thus, various attempts were made to detoxify the venom toxins. This has been done by chemical means such as treatment with formaldehyde (28), glutaraldehyde (17, 29), by iodination (30), and by physical means using X-irradiation (31), UV light (32) and gamma irradiation (33). It is relevant to note that the detoxification reactions invariably involve either modification of the ‘active site’ or otherwise alter the conformation of the toxins, thereby rendering them inactive. Consequently, owing to the modifications introduced in the structure of these toxins, the antibodies generated against these detoxified toxins usually failed to recognize and neutralize the native toxins leading to low potency of the antivenom. Furthermore, some of these detoxification reactions, e.g. glutaraldehyde polymerization (29) and iodination (34), are difficult to control and optimize especially when different toxins are involved, as occurs in the preparation of polyspecific antivenoms. Finally, immunization at high doses of (detoxified) venom could lead to immune tolerance (35, 36).

The low neutralizing potency of antivenoms was also thought to be due to the low molecular mass of elapid α-neurotoxins which might be associated with their low immunogenicity (37). Thus, various studies were made to conjugate the toxins to macromolecules or immunogenic carrier proteins e.g. bovine serum albumin (BSA) or tetanus toxoid. An example is the conjugation of toxins to cellulose particles that had been oxidized with sodium metaperiodate (SOC). These conjugates were found to increase anti-neurotoxin antibody titers 2.0-2.5 fold relative to the native toxin (38). However, these types of reactions (detoxification and conjugation/polymerization) on the venoms have not yet been applied to commercial antivenom production.

To identify the key factor(s) involved in the production of potent antivenoms, we carried out a study using nine immunogens prepared from the α-neurotoxin of a cobra venom (*Naja kaouthia* toxin 3, NK3). These immunogens included the crude venom, the purified toxin, various carbodiimide conjugates, and polymers obtained from controlled polymerization by glutaraldehyde or formaldehyde. These immunogens were tested in rats using Freund’s adjuvants (29). It was shown that pure NK3 toxin elicited comparable specific antitoxin antibody titers as that of the crude venom which suggested that the toxin was immunogenic, and that ‘antigenic competition’ (39), if present, was not an important factor in the antibody response against the elapid toxins. The results also showed the absence of any immunosuppressive component in the crude venom (40) that could reduce the antibody response against the toxin. Thus the experiment clearly underscored that the elapid α-neurotoxins are capable of inducing a good antibody response in spite of the fact that they are of low molecular mass.

This conclusion is supported by our recent finding that the amino acid sequences of the α-neurotoxins contain T cell epitopes that are required for binding to major histocompatibility complex (MHC) class II proteins. The T cell epitope–MHC complex then interacts with a CD4+T cell receptor (TCR). The activated CD4+T cell then initiates a sequence of events leading to the production of toxin specific antibodies. The lack of T cell epitope abrogates activation of CD4+T cells and T cell dependent antibody responses (41, 42). In the case of the 71 residue α-neurotoxin from the cobra *Naja siamensis*, we used an online program “IEDB analysis resource” (43) to predict the T cell epitopes for human HLA (no information on horse MHC II was available in IEDB). The two T-cell epitopes are predicted to be in amino acids 1-9 (medium score epitope) and 28-36 (high score epitope). The fact that α- neurotoxins contain high score T cell epitopes and thus would be expected to be immunogenic, raised the question as to why antivenoms against the elapids are usually of low potency. There should be some other parameters employed in the immunization, i.e. dose, adjuvant, route of administration, volume, frequency (44), that contributed to the observed low potency.

Experiments on the immunogenicity of various derivatives of α-neurotoxins described above were carried out in rats using a variety of adjuvants including Freund’s adjuvants. However, these adjuvants have been shown to cause granuloma and sterile abscesses at the site of immunogen injection (45–47). Consequently, their use in horses was discouraged (48). Thus, many antivenom producers use other adjuvants (bentonite, squelene/Aracel A, aluminum salts, sodium alginate etc.) in their antivenom production. The Thai Red Cross at Queen Saovabha Memorial Institute (QSMI) used bentonite as the adjuvant in horses.

We therefore carried out a comparative study on some of the *N. kaouthia* toxin immunogens using three different adjuvants (IFA, bentonite and squalene/Arlacel A). The results showed that only IFA gave a good specific antitoxin antibody response (49). Thus, it was concluded that the low antibody response normally observed in PDAV production in horses was mainly due to the ineffective adjuvant used and not necessarily to the low immunogenicity of the toxins. Since co-administration of the immunogen with an effective adjuvant is an essential requirement in antibody production (36, 41), it is critical that the most effective adjuvant is used in the horse. Therefore, to improve the effectiveness of PDAV production, it was necessary either to find new and better adjuvants, or to find a way to use CFA/IFA safely in horses to avoid the adverse reactions previously observed. The ineffectiveness of the therapeutic antivenoms available combined with a severe shortage in Thailand at the time created a critical situation that led us to choose Freund’s adjuvants since they have an excellent record of immunostimulatory effect as compared to other adjuvants (50–52).

Complete Freund Adjuvant (CFA) is a water-in-oil suspension containing purified light paraffin oil and mannide monooleate, a surfactant, as emulsifier. It also contains heat-killed dried *Mycobacterium tuberculosis*. The Incomplete Freund Adjuvant (IFA) contains the same ingredients but without the Mycobacterium. With CFA, the hydrophilic and amphipathic snake toxins reside in the aqueous phase, which keeps them in their native conformation (53). The water-in-oil preparation serves as a depot for slow and continuous release of the venom antigens from the injection site for prolonged stimulation of antibody producing cells. It also protects the immunogen from rapid proteolytic degradation and elimination, resulting in the production of high levels of antibody by the host. The mineral oil component of the antigen adjuvant emulsion serves as a vehicle for antigen transport throughout the lymphatic system to immune effector cells and promotes interaction with antigen-presenting cells like dendritic cells. In CFA, the mycobacterial cell wall contains lipoprotein, lipomannans and lipoarabinomannans that interact with Toll-like receptor-2 (TLR-2), as well as TLR-4 and TLR-6 (54–57). This provides immunostimulation by recruiting, activating, and enhancing differentiation of the cells of the immune system (58). It has been shown that CFA and IFA have served with unsurpassed record in the stimulation and production of high titers, high affinity and high avidity antibodies (50, 51) and are useful for low molecular weight antigens (59). Moreover, these adjuvants can be produced in large volume with high consistency and inexpensively. Thus, an immunization protocol that allows the safe use of CFA/IFA must be found.

### The Use of Freund’s Adjuvants in Horses and the ‘Low Dose, Low Volume Multi-Site’ Immunization Protocol

After extensive inquiries and discussions with some PDAV producers, it was concluded that the severe adverse reactions in horses resulting from CFA injection were due to the injection of a large volume of immunogen emulsified in CFA at one single anatomical site (47, 60–62). The inflammation at the large injection area inevitably caused skin rupture that, under non-aseptic conditions, caused severe infection that in some cases could result in death. It was hypothesized that the lesions produced by CFA could be reduced or eliminated by minimizing both the total injection volume and the volume injected at each site.

**Figure 1:**
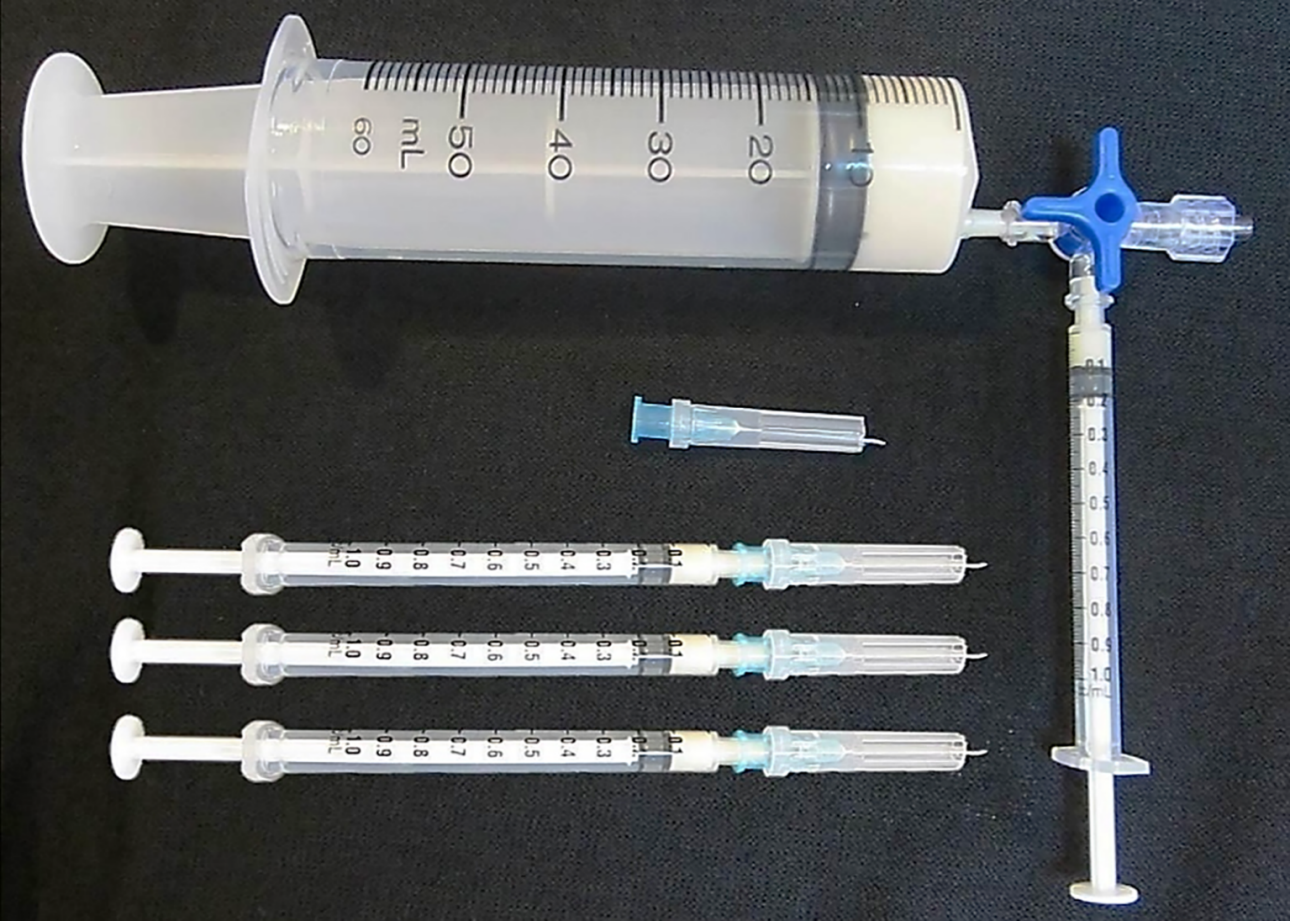
The modified tuberculin syringes used to deliver 0.1 ml of Freund’s adjuvant emulsified immunogen into the horse subcutaneous site at the depth of 2-3 mm from the skin surface according to the ‘low dose, low volume multi-site’ immunization. Please see details in (66).

A simple and novel immunization protocol termed ‘low dose, low volume, multisite’ immunization was therefore proposed and tested in horses (63, 64). This immunization protocol involves subcutaneous injection of the CFA emulsified immunogen in small volumes (50-200 µl/site) carrying a very low venom dose (about 1-2 mg of venom in total/horse) around the neck at approximately 20 sites. Because of the low volume injected at each site, the local reaction is mild, and when the preparation of immunogen and the injection were performed aseptically, no infection or skin rupture occurred (63, 64).The injection is made subcutaneously (2-3 mm depth from the skin surface) in the epidermis where the dendritic cells, the most potent antigen presenting cells, are located (65). Furthermore, the injections are made around the neck area of the horse where the majority of the lymph nodes are situated. This ensures maximum exposure of the immunogen to the lymphocyte traffic. The low volume used at each of the 20 injection sites also increases the total exposed surface area of the droplets containing the immunogen by 2.4 times when compared to injection of the total volume at one single site, assuming the droplets are spherical. This results in increased exposure of the immunogen to the stellar shape dendritic cells. The simple immunization procedure can be easily carried out (taking about 2-3 minutes to inject 20 sites per horse) using slightly modified tuberculin syringes (**Figure 1**). Using this procedure the reaction at the injection sites was mild or absent (63, 64). This protocol was included and recommended in the WHO Guidelines for antivenom production and control (66).

This immunization protocol has allowed the safe use of Freund’s adjuvants. It has been repeatedly shown to induce high specific antibody titers in horses (41, 67–69).

It should be noted that the very low venom/toxin dose used for the immunization not only reduces the cost because of the lower amount of venom used but, more importantly, also stimulates the production of high affinity antitoxin antibody thus increasing PDAV potency (67–69).

This novel immunization protocol has resulted in the production of highly potent antiserum (2-4 fold increase in potency) against *N*. *kaouthia* venom (63). It reduces the time required for a horse to reach maximum antibody titers to about 6 to 8 weeks instead of several months. It reduced the amount of venom immunogens to just 10% of what was previously used; and it increased the percentage of responder horses from about 60% to 100% (70). These improvements have resulted in vast increase in antivenoms production by QSMI (70) with enough surpluses for export to neighboring countries. Furthermore, this development is partly responsible for the decrease in the country’s annual snakebite death from a few dozens to mostly nil in recent years (Snakebite in Thailand, Annual Epidemiological Surveillance Report, Ministry of Public Health).

The introduction of this novel immunization protocol has also resulted in the successful production of two potent polyspecific antivenoms. Based on a ‘Syndromic strategy’ (9), one polyspecific antivenom is against three neurotoxic venoms: *N*. *kaouthia* “Thai monocellate cobra”, *Bungarus fasciatus* “Banded krait” and *Ophiophagus hannah* “The King cobra” (64) while another is against three hematotoxic venoms: *Cryptelytrops albolabris* “White lipped pit viper”, *Calloselasma rhodostoma* “Malayan pit viper”, and *Daboia siamensis* “Russell’s viper” (69). These polyspecific antivenoms are currently produced commercially by The Thai Red Cross at QSMI using the ‘low dose, low volume multi-site’ CFA immunization protocol. Recently, the polyspecific anti-neurotoxic antivenom produced by QSMI also includes the venom of *Bungarus candidus* “the Malayan krait”.

## Paraspecificity of PDAV and the Production of Pan-Specific Antivenom Against Neurotoxic Venoms

The production of poly specific AVs using the “low dose, low volume, multisite” immunization protocol has led to improvements in treatment due to reduced production costs and increased effectiveness of antivenoms. Interestingly, these two polyspecific AVs have been shown to significantly cross-neutralize various medically important hematotoxic and neurotoxic venoms of snakes distributed in Southeast Asian and South Asian countries (71–74). Moreover, it has been observed that many other polyspecific AVs offer immunochemical cross reactivity with heterologous venoms from various species (6, 75–80).

It would be highly desirable if a PDAV can be produced to cover dozens of related venoms which are medically important to people in several countries or regions. Such a ‘pan-specific’ PDAV should be very useful to a large number of snakebite victims. However, the upper limit of venoms used as immunogens for polyspecific antivenom production is only about 5-6 venoms. When higher number of venoms are used in the immunization, lower antibody titers against some or all the venoms are obtained.

## A Simple and Novel Immunization Strategy Using a ‘Diverse Toxin Repertoire’ as Immunogen and the Production of Pan-Specific Antivenom Against Elapid Snake Venoms

From the above discussion, it was noted that while some of the monospecific antivenoms exhibited no or very narrow cross reactivity (5–7, 81), polyspecific antivenoms prepared from various laboratories showed wide paraspecificity (71–80). It seemed that the number of heterologous venoms neutralized by a polyspecific antivenom is greater than the sum of heterologous venoms neutralized by the antivenoms prepared separately as monospecific antivenoms. These observations suggested that the numerous antibodies in a polyspecific antivenom somehow act cooperatively to cross neutralize heterologous toxins, resulting in wider paraspecificity of the antivenom. This can happen if two or more heterologous antibodies bind, even weakly, to a target toxin and together enable cross-linking to form lattice and neutralization which otherwise could not occur. This would be a ‘positive cooperative’ effect of the heterologous antibodies against a heterologous toxin. If this conjecture is true, it should be possible to prepare wider paraspecific antivenoms by increasing the number of venoms used in the immunization.

With the aim of producing a pan-specific PDAV against elapid venoms of Asia, we therefore proposed and tested a simple and novel immunization protocol using a ‘diverse toxin repertoire’ consisting of several neurotoxic venoms as immunogen (82). The ‘diverse toxin repertoire’ was obtained from toxin fractions of 12 neurotoxic venoms of Asian origin. These toxin fractions were prepared by ultrafiltration of the venoms to remove toxicologically-irrelevant high molecular mass and highly immunogenic venom proteins. The fractions were individually shown to contain the lethal toxins (α-neurotoxins and β-neurotoxins) and total lethal activity of the venoms. The mixture of these toxin fractions was used to immunize horses at very low doses (about 12 μg of each toxin fraction) using the ‘low dose, low volume multisite’ protocols (63). It was found that the horse antiserum could neutralize 11 homologous and 16 heterologous neurotoxic venoms from elapids of Asian and some African countries (82). Thus the pan-specific PDAV could offer broad cross neutralization of venoms from different and geographically separated snakes and could benefit a large number of snakebite victims. The rationale of the novel immunization strategy was discussed previously (83). The result of this study is a proof of concept of the ‘diverse toxin repertoire’ immunization strategy in the production of pan-specific antivenom against neurotoxic venoms. It also indicates that it should be possible to produce a universal PDAV against the elapid snakes of the world.

## A Quest for Universal PDAV Against all the Neurotoxic Elapid Snakes

To further test the concept of the ‘diverse toxin repertoire’ immunization strategy, we assayed the ability of the pan-specific PDAV to inhibit the venoms of a variety of elapids from different continents. It was shown that the pan-specific PDAV could effectively neutralize at least 36 neurotoxic venoms of 10 genera and from 4 continents including sea snakes from both Australia and the Arabian Sea (83).

These results suggest that universal antivenom against all elapid snakes is possible if the ‘diverse toxin repertoire’ is modified to include a few more neurotoxic venoms. The bases for our idea are as follows.

a). Most of the elapid venoms contain α–neurotoxins and some also contain the highly lethal β-neurotoxins. For simplicity, the discussion will be confined only to the α–neurotoxins. All of the elapid α–neurotoxins are highly lethal and are responsible for most of the deaths caused by a large number of elapid species. They have high amino acid sequence homology with one another and all share the same mechanism of toxicity in that they bind specifically to the α–subunits of nAchR at the motor endplate in the neuromuscular junction (26, 84, 85). Thus all these toxins are structural and functional homologs. These toxins, although previously believed to be poorly immunogenic, are in fact able to induce high affinity neutralizing antibody (41, 67). This is supported by the fact that they all contain high score T helper epitopes in their molecular sequences as discussed above.
b). The horse antibody repertoire is vast and far exceeds the epitope repertoire of all the world’s elapid α-neurotoxins. Thus the horse is capable of producing specific antibodies against any elapid α-neurotoxin. This conclusion is based on the following information and calculation.

### The Repertoire of the Elapid α–Neurotoxin Epitopes

Given their small molecular size and constraints imposed with the formation of a biologically active conformation, it is likely that each α–neurotoxin contains a relatively small number of dominant epitopes on its surface, with each epitope made up of about 12 amino acid residues (86, 87). The average accessible surface area of an epitope is about 846.59 ± 278.87 sq Å (88). The total accessible surface area of the 71 amino acid residue α-neurotoxin of *N. siamensis*, venom (PDB 1CTX; alpha-cobratoxin from *Naja siamensis*) is calculated to be about 5,206 sq. Å using a program described by Ribeiro et al. (89). The number of non-overlapping epitopes on the α-neurotoxin surface is therefore about 6 epitopes per toxin.

The family Elapidae comprises 382 species (www.reptiledatabase.org). Assuming that each of these shows three geographic variations regarding their α-neurotoxins structure, this will give 1,146 (382x3) α-neurotoxins amino acid sequences. If each elapid produces an average of three different α-neurotoxin isoforms (83), this will give a total of 3,438 elapid neurotoxin isoforms. If each isoform has six non-overlapping epitopes, a total of about 2.06 x10^4^ elapid α-neurotoxin epitopes would exist in nature. This number is probably overestimated since some of the epitopes from homologous toxins are conserved and similar for structural and functional reasons. However, suffice is to say that the total number of different epitopes of the world elapid α-neurotoxins is finite and in the range of tens of thousands.

### The Horse Antibody Repertoire

On the opposite end of the antigen-antibody interaction is the antibody paratope. The diversity of the antibody paratopes generated spontaneously in a large animal, e.g., human and horse, is enormous. Due to the random immunoglobulin genes rearrangement, it has been estimated that 10^15^-10^18^ of naïve antibody specificities could be generated (90, 91). In another study, it was estimated that the total potential repertoire in human is immense at 10^26^ different antibody specificities (92). However, with new immature B cells being produced at the rate of about 10^9^ per day and the number of circulating peripheral naïve mature B-cells at any one time is about 10^9^ (93), a repertoire size of naïve antibodies in human is thought to be about 10^12^ specificities (94). Moreover, this repertoire of naïve antibodies is expanded exponentially by somatic hypermutation after antigen encounter (95). This number is thought to be adequate to handle about 1400 potential pathogenic species thought to be infectious to humans (96).

From the above calculation, the total number of elapid α-neurotoxin epitopes is about 2.06 x10^4^. Therefore, the naive antibody sequences present each day in the horse should be enough to recognize and bind to all the epitopes of elapid α-neurotoxins of the world.

c) It is likely, therefore, that when a horse is immunized with the ‘diverse toxin repertoire’ from venoms of numerous snakes, there will be enough B-cells with antibody paratopes/specificity generated against this repertoire. Furthermore, these antibodies should include a large number of those capable of cross reacting with other heterologous toxins. That this is most likely the case is supported by our previous results (83). When the horse was immunized with the toxin fractions of 12 Asian elapids of only 6 species and 2 genera (*Naja* and *Bungarus*), the antiserum was shown to neutralize 36 venoms of 28 species and 10 genera from 4 continents.

It should be mentioned that among the 37 elapid venoms tested, only the heterologous venom of *Dendroaspis angusticeps* was not neutralized by the pan-specific antivenom (83). The lethal toxins of this venom have not been identified but are thought to act synergistically (97, 98). Thus, if the toxin fractions of this mamba together with other selected WHO Category 1 elapid venoms (66) from various continents are included in the immunization mix, it is most likely that a PDAV with paraspecificity against all elapid neurotoxic venoms can be produced.

A universal PDAV against neurotoxic snake venoms would be analogous to the equine anti-rabies and anti-tetanus sera, in the sense that it could be used in a wide geographical range. Using the facilities already available in most antivenom producers, the production could be implemented within a relatively short time and without additional investment. It could be produced in large volume and, with the economy of scale, it could be produced at relatively low cost and be affordable to low income snake bite victims of the world. Last but not least, the availability of a universal PDAV eliminates the need for species identification of the culprit elapids.

## Advantage and Challenges of The ‘Diverse Toxin Repertoire’ Immunization Strategy

One advantage of the ‘diverse toxin repertoire’ immunization strategy is that it generates unprecedented wide paraspecificity against at least three dozen elapid venoms and possibly against all elapid venoms having α-neurotoxins as lethal components. Moreover, the procedures involved are very simple. For example, the preparation of the toxin fractions, the immunization and the antibody fractionation can be readily carried out using the existing facilities of most current antivenom producers. Ultrafiltration can be used to purify the neurotoxic fractions because the requirement is to obtain a mixture of lethal toxins with all its isoforms, rather than any single purified toxin. The fractionation process of antibody IgG or F(ab’)2 can be carried out using the equipment for routine PDAV production in antivenom manufacturing laboratories. Furthermore, the production time should be shorter than that required to produce several monospecific or polyspecific PDAVs. The cost of producing one universal antivenom might be lower than that for several polyspecific antivenoms. However, detailed analysis on the cost of all the production steps is necessary to make a valid comparison.

It should be mentioned that although a polyspecific PDAV can neutralize many venoms, its potency (ED50 or Effective Dose50) against different neurotoxic venoms may vary and thus different dosages of the PDAV may be required for treatment of envenomation by different elapids. However, this is quite normal in the treatment of snakebite envenomation because the antivenom dose administered to the patient depends on the severity of the case, which largely depends on the amount of venom delivered by the snake. This in turn depends on many parameters; for example, the sizes of the snake and of the victim, the toxicity of the venom, the site of the bite, the time lapse between the bite and the treatment, etc. Thus protocols are developed to establish the dose that needs to be administered.

However, it is likely that the paraspecific potency of the PDAV against some heterologous venom(s) may be low and may pose a clinical problem. In these cases, the neutralizing potency against these venoms can be improved by modifying the immunization schemes and/or by antibody fractionation in the following ways. First, during the immunization, the heterologous venoms that are poorly neutralized could be included in the ‘diverse toxin repertoire’ immunogen and thus serve as homologous venom antigens so as to increase the antibody titers against them. The neutralizing potency against some venom can also be enhanced by booster injections with the toxin fractions of only these poorly neutralized venoms. These immunization schemes have been shown to work well in the production of the polyspecific antivenoms in Thailand. Second, during antibody fractionation, the neat horse serum can be fractionated by salt precipitation to obtain about 2 fold increases in neutralizing potency (99). Subjecting this refined globulin fraction to α-neurotoxin affinity chromatography could result in a further 10 to 12 fold increase in potency (100). Thus, a 20 fold increase in neutralizing potency could be achieved by antibody fractionation. The combination of optimized immunization and antibody fractionation will result in substantial increases in the neutralizing potency of the antivenom over that of the antiserum.

With these modifications and optimization, it is likely that universal PDAV against elapid neurotoxic venoms can be produced and used for the treatment of envenomation by elapid snakes.

## Conclusion

Plasma-derived antivenom (PDAV) is still the mainstay of the current therapies for snakebite victims. There are some drawbacks to PDAV and this has led to attempts to produce ‘new generation’ antivenoms. However, this is likely to take some time until these new therapies reach the clinical trial stage. Consequently, improvements to PDAVs that lead to the production of universal PDAV against the world elapid venoms would be of immediate benefit. The production of a pan-specific PDAV against at least three dozen neurotoxic venoms from four continents has been achieved through a simple and novel immunization strategy using a ‘diverse toxin repertoire’ as immunogens. The ‘diverse toxin repertoire’ was made up of toxin fractions of numerous elapid venoms. The strategy has resulted in unparalleled wide paraspecificity. With careful selection of toxin fractions of elapid venoms to serve as immunogens together with an optimized immunization scheme and antibody fractionation, it is most likely that a universal PDAV with high neutralizing potencies against elapid venoms can be produced. Such a PDAV is analogous to the anti-rabies and anti-tetanus antitoxins that are produced for use worldwide. Universal PDAVs can be produced in large volume which, with the economy of scale, should be more affordable to poor snakebite victims in many parts of the developing world and save numerous lives before ‘new generation’ antivenoms become available.

## Author Contributions

The author confirms being the sole contributor of this work and has approved it for publication.

## Funding

The research work of the author was supported by research grants from Mahidol University, National Research Council of Thailand, The Thailand Research Fund, National Science and Technology Development Agency of Thailand (grant no. CPT 89-1-05-143), The United States Agency for International Development (grant no. 936-5542-G-00-5079-00) and Chulabhorn Research Institute (grant no. IM 2011-01 to KR).

## Conflict of Interest

The author declares that the research was conducted in the absence of any commercial or financial relationships that could be construed as a potential conflict of interest.
